# A Realistic Validation Study of a New Nitrogen Multiple-Breath Washout System

**DOI:** 10.1371/journal.pone.0036083

**Published:** 2012-04-27

**Authors:** Florian Singer, Birgitta Houltz, Philipp Latzin, Paul Robinson, Per Gustafsson

**Affiliations:** 1 Division of Respiratory Medicine, Department of Paediatrics, University Children's Hospital of Bern, Bern, Switzerland; 2 Department of Clinical Physiology, East Hospital, Sahlgrenska University Hospital, Gothenburg, Sweden; 3 Department of Paediatrics, The Children's Hospital at Westmead, Sydney, Australia; 4 Department of Paediatrics, Central Hospital, Skoevde, Sweden; University of Tübingen, Germany

## Abstract

**Background:**

For reliable assessment of ventilation inhomogeneity, multiple-breath washout (MBW) systems should be realistically validated. We describe a new lung model for *in vitro* validation under physiological conditions and the assessment of a new nitrogen (N_2_)MBW system.

**Methods:**

The N_2_MBW setup indirectly measures the N_2_ fraction (F_N2_) from main-stream carbon dioxide (CO_2_) and side-stream oxygen (O_2_) signals: F_N2_ = 1−F_O2_−F_CO2_−F_Argon_. For *in vitro* N_2_MBW, a double chamber plastic lung model was filled with water, heated to 37°C, and ventilated at various lung volumes, respiratory rates, and F_CO2_. *In vivo* N_2_MBW was undertaken in triplets on two occasions in 30 healthy adults. Primary N_2_MBW outcome was functional residual capacity (FRC). We assessed *in vitro* error (√[difference]^2^) between measured and model FRC (100–4174 mL), and error between tests of *in vivo* FRC, lung clearance index (LCI), and normalized phase III slope indices (S_acin_ and S_cond_).

**Results:**

The model generated 145 FRCs under BTPS conditions and various breathing patterns. Mean (SD) error was 2.3 (1.7)%. In 500 to 4174 mL FRCs, 121 (98%) of FRCs were within 5%. In 100 to 400 mL FRCs, the error was better than 7%. *In vivo* FRC error between tests was 10.1 (8.2)%. LCI was the most reproducible ventilation inhomogeneity index.

**Conclusion:**

The lung model generates lung volumes under the conditions encountered during clinical MBW testing and enables realistic validation of MBW systems. The new N_2_MBW system reliably measures lung volumes and delivers reproducible LCI values.

## Introduction

Multiple-breath inert gas washout (MBW) tests are used to assess uniformity of ventilation distribution in infants, children, and adults [1]. Both global indices of ventilation inhomogeneity such as the lung clearance index (LCI) and specific indices of non-uniform gas mixing in the *conducting* and *acinar* airway zones (S_cond_ and S_acin_) are frequently reported [2]–[6]. The accuracy of these indices relies directly or indirectly on correctly measured functional residual capacity (FRC). Therefore current guidelines for lung function testing recommend *in vitro* FRC measurements over the full range of lung volumes in question at various respiratory frequencies [7], [8].

FRC measurement validation is commonly assessed using calibration syringes to simulate FRC and tidal breathing washout [9]. However, *in vivo* accuracy of FRC measurements depends on the individual performance under physiological conditions of the gas and flow sensors used, ignored by the syringe. Given the increasing interest in MBW as a clinical outcome measure there is an urgent need for improved validation models which incorporate conditions encountered during clinical testing (*i.e.* variations in temperature, pressure and humidity over the breath cycle).

This report describes (i) the development and utility of a lung model incorporating simulated body temperature, pressure and water vapor saturation (BTPS) conditions, and (ii) the *in vitro* and *in vivo* performance of a commercial N_2_MBW system (Exhalyzer D®, Eco Medics AG, Duernten, Switzerland). Primary outcome was the accuracy and precision with which FRC can be generated and measured *in vitro* and the reproducibility of FRC *in vivo* in healthy adults. Secondary outcomes were the reproducibility of ventilation inhomogeneity indices and their correlation in healthy adults.

## Methods

### Nitrogen multiple-breath washout setup

We used an unmodified open-circuit N_2_MBW hardware and software package (Exhalyzer D® and Spiroware® 3.1, Eco Medics AG) for all recordings, and an in-house customized software based on TestPoint™ (Capital Equipment Corp, Billerica, MA, USA) for off-line data processing and analyses. Flow was measured using a mainstream ultrasonic flowmeter and used to derive tidal volumes [10]. Gas concentrations were measured by a side-stream laser O_2_ sensor (Oxigraf, Inc, Mountain View, CA, USA) and a main-stream infra-red CO_2_ sensor (Capnostat® 5, Respironics Novametrix LLC, Wallingford, CT, USA). In this device, F_N2_ is measured indirectly based on *Dalton*'s law of partial pressures: F_N2_ = 1−F_O2_−F_CO2_−F_Argon_ (F_Argon_ = F_N2_ * 0.00934/0.78084). The F_Argon_ (0.00934) is treated as a fixed proportion of the F_N2_ assuming similar washout during N_2_MBW. Daily two-point calibration and verification of the flow and O_2_ sensors, and zero calibration of the CO_2_ sensor were performed. The O_2_ sensor has a slower 10–90% response time (140 ms) than the CO_2_ sensor (55 ms). To align their signals, a speeding algorithm was applied to the O_2_ signal reducing its response to approximately 110 ms [11], [12]. Gas signals were synchronized to the flow signal using the re-inspired post-capillary dead space to produce a step response in CO_2_ and O_2_. The gas signal vectors were time shifted to the point in time when the post-capillary dead space had been inhaled such that a 50% change in gas signal deflection then occurred. This was repeated over a minimum of ten washout breaths and median “delay times” for CO_2_ (50 ms) and O_2_ (565 ms) were used for signal alignment. Quality of superimposition of the inverted O_2_ signal on the CO_2_ signal was assessed visually.

**Figure 1:**
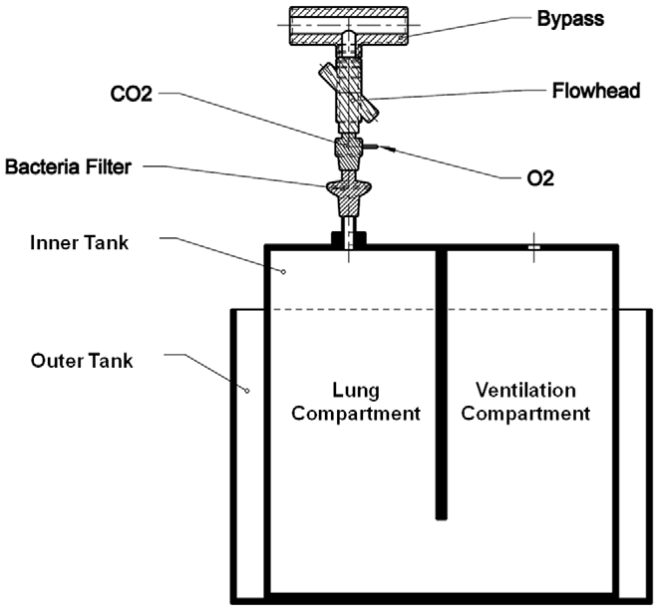
Schematic drawing of the lung model. The lung and ventilation compartments are partly filled with distilled water, their volumes are 6.94 L and 11.72 L, respectively. The outer chamber's volume is 67.73 L, filled with distilled water and constantly heated to 37°C. The dashed line reflects the water level determining tidal volume and functional residual capacity (FRC) at in- and expiratory end-tidal levels. FRC is the volume of air contained in the model at end-tidal expiration. One mm on the tape measure corresponds to 18.7 mL volume. FRC range is 0.5–4.2 L. O_2_ = sampling point of the side-stream oxygen sensor. CO_2_ = sampling point of the main-stream carbon dioxide sensor. Flowhead = main-stream ultrasonic flowmeter.

Lung (model) resident F_N2_ was washed out using 100% O_2_ applied via open circuit at either 200 mL/s for 100–400 mL FRCs or at 1000 mL/s for 500–4200 mL FRCs and *in vivo*, respectively (Figure 1). These bypass flows were chosen to exceed maximum tidal inspiratory flows and to minimize rebreathing of CO_2_ or N_2_. For respective lung volumes (Table 1) we used post-capillary dead space reducers (infant set 1, preschool set 2, adult set 3) and hygienic inserts (Spirette) provided by the manufacturer (Eco Medics AG). These reduced equipment related post-capillary dead space (volume between CO_2_/O_2_ sampling point and bypass) to 1.5 mL, 16 mL, and 26.9 mL, respectively, as measured by water displacement. Bacteria filters (air eco slimline, Vickers Ind Est, LA, UK) had 30 mL dead space and were used for preschool and adult sets. Accordingly, pre-capillary dead space (volume between lung compartment top and CO_2_/O_2_ sampling point) was 37.9 mL in the large model and 3 mL in the small model, respectively. Apparatus resistance was measured by a pressure transducer (Timeter RT200, Allied Healthcare Products Inc., MO, USA). We applied pure O_2_ and increased flows stepwise between 0–200 mL/s for the infant set, 0–500 mL/s for the preschool set, and 0–770 mL/s for the adult set with bacteria filters in place. Maximum resistances were 0.03, 0.06, and 0.19 kPa/L*s, respectively, and complied with the recommendations of previous standards [8], [13].

**Table 1:** Study protocol for the lung model.

Lung model	DSR	Trial (n)	FRC (mL)	V_T_ (mL)	RR (min^−1^)
Small	1	7	100–400	30–80	20–35
Large	2	10	500–2000	150–540	12–29
Large	3	33	2000–4200	190–880	10–29

Functional residual capacity (FRC), tidal volume (V_T_), and respiratory rate (RR) generated in triplets are displayed as ranges. Respective dead space reducers (DSR 1–3) were applied to reduce post-capillary dead space to 1.5 mL, 16 mL, and 26.9 mL, respectively. Mean (range) intra-test coefficient of variation (CV) of generated nominal FRCs was 0.1 (0.0–1.8)%.

### The lung model

The framework of the lung model was constructed from acrylic glass (Soloplex, Tidaholm, Sweden) and consisted of two rigid chambers: An inner chamber divided into two communicating compartments (via their lower aspect) termed the lung and the ventilation compartment, and an outer chamber (Figure 1). Two different size models were constructed to allow for both infant (100 to 400 mL) and children/adult (500 to 4200 mL) lung model volumes. The inner chamber was filled with distilled water until the desired FRC was achieved, measured as the end-expiratory water level using a transparent vertical tape measure fixed to the lung compartment. FRC volume was determined geometrically from known dimensions: one millimeter corresponded to 18.7 mL in the large and to 4.8 mL in the small lung model. The lung model was placed inside a water tank which served to heat the water in the inner chamber to 37°C, monitored using a thermostat. To simulate various breathing patterns, a bi-level positive airway pressure ventilator (Vivo 30, Breas Medical AB, Mölnlycke, Sweden) for the large lung model, or a 100 mL calibration syringe (Hans Rudolph Inc, Shawnee, KS, USA) for the small lung model was connected to the top of the ventilation compartment and exerted hydraulic pressure transmitted through to the lung compartment. During ventilation, temperature and humidity were measured every third FRC trial inside the lung compartment using a thermocouple thermometer (BAT-12 Microprobe Thermometer, Physitemp Instruments, Inc. New Jersey, USA) and a monolithic humidity sensor (HIH-4602-L-CP, linear humidity sensor, Honeywell, Minneapolis, USA).

### *In vitro* study

Static signal linearity of the N_2_MBW system was assessed over the full range of O_2_, CO_2_, and N_2_ fractions encountered during N_2_MBW testing and performed at 37°C with 100% relative humidity: By increasing F_O2_ stepwise (60 steps), F_O2_ ranged from 15–100%, F_CO2_ from 0–6%, and F_N2_ from 0–79%. The reference for measured gas concentrations was a respiratory mass spectrometer (AMIS 2000; Innovision A/S, Odense, Denmark).

*In vitro* assessments of the N_2_MBW system performance were undertaken as triplicate FRC measurements (n = 150) across 50 different nominal FRCs (100 to 4200 mL), over two days. Ventilator pressures in the large model or syringe stroke volumes in the small volume were chosen to achieve physiological tidal volumes (V_T_), V_T_ over FRC ratios (V_T_/FRC), and respiratory rates (RR) for each FRC setting (Table 1). To assess the possible influence of different F_CO2_ on FRC measurement accuracy, the lung compartment was washed-in with either ambient air (0.05% CO_2_) or CO_2_ enriched air (n = 45, Carboair®, Aiolos Medical, Karlstad, Sweden) prior to N_2_MBW. Carboair® contains 5% CO_2_ and balance air.

### *In vivo* study

Thirty-two healthy adults performed N_2_MBW on two test occasions within a three week period. All subjects had a standardized interview on respiratory health. Inclusion criteria were adults aged 19 to 70 years with a smoking history less than five pack-years, no history of acute or chronic airway disease, and no on-going medication potentially affecting lung function. N_2_MBW was performed in the sitting position using a nose clip with the dead space reducer (set 3), hygienic insert, and bacterial filter in place. N_2_MBW was done in triplets with between-test intervals exceeding the washout time. The subjects were instructed to breathe regularly with relaxed expirations. The washout phase was terminated once end-tidal F_N2_ was less than 1/40^th^ of the starting F_N2_ for at least three breaths.

### Ethics statement

The *in vivo* study was approved by the Ethics Committee of the University of Gothenburg Sweden. We obtained informed consent from all participants involved in the study.

### Nitrogen multiple-breath washout outcomes

FRC was calculated as *net* expired N_2_ volume (expired N_2_ volume minus re-inspired N_2_ volume) divided by the difference of F_N2_ at start of MBW minus F_N2_ at end of MBW. Pre-capillary dead space was subtracted from FRC such that the reported FRC corresponds to the volume of the inner compartment of the lung model or FRC at the airway opening in the *in vivo* recordings. Indices of ventilation inhomogeneity were calculated from *in vivo* N_2_MBW trials. Because LCI, S_acin_, and S_cond_ relate inversely to ventilation efficiency, their values increase with increasing ventilation inhomogeneity. LCI was calculated as cumulative expired gas volume (CEV) required to reduce F_N2_ to 1/40^th^ of the starting F_N2_, divided by FRC. Two phase III slope (S_III_) indices, S_cond_ and S_acin_, were calculated. Automated S_III_ fitting over 50–95% of expired volume was performed for each breath and manually adjusted to exclude phase II or IV from the linear regression fit. S_III_ was normalized (Sn_III_) by dividing S_III_ by the mean F_N2_ over S_III_ and multiplying S_III_ with V_T_, and averaged per breath from the three N_2_MBWs [14]. Mean Sn_III_ per breath was then plotted against the corresponding mean lung volume turnover (TO = CEV/FRC) for each breath. S_cond_ is defined as the rate of Sn_III_ increase between lung volume turnovers 1.5 and 6.0. S_acin_ is defined as the first breath Sn_III_ value minus the convection-dependent inhomogeneity contribution to this value [15].

### Statistics

Linearity of sensors compared to mass spectrometry was assessed using uni-variable linear regression and respective signal offsets (linear model intercept) and gains (linear model slope) with their 95% confidence intervals (CI) were reported. Intra-test variability of both model and measured FRCs as generated by the model and measured by the N_2_MBW setup, respectively, was calculated as intra-test coefficient of variation (CV = SD/mean*100). Accuracy of FRC measurements was expressed as (i) absolute (mL) difference (difference between measured FRC minus model FRC), (ii) relative (%) difference (difference*100 divided by model FRC), and as (iii) relative error (square-root of the squared relative difference). Acceptable upper limit was 5% error according to current infant lung function standards [7]. We reported coefficient of repeatability (CR) calculated as 1.96*SD of differences between paired measurements [16]. The CR estimates the 95% range of technical variability due to measurement error *in vitro* or the technical and physiological between-test variability *in vivo*. We assessed *Bland Altman* plots [16], and the relation of error with FRC, breathing pattern, different lung model gases, and between tests using uni- and multi-variable linear regression models and paired *t*-tests. P-values<0.05 were considered statistically significant and all analyses were done using Stata™ (Stata Statistical Software: Release 11. College Station, TX: StataCorp LP).

## Results

### Lung model performance

The lung model successfully generated 146 (97%) out of 150 FRCs (range 100–4174 mL) under BTPS conditions. Mean (SD) of temperature and humidity were 35.9 (1.3)°C and 97.9 (1.0)%, respectively, obtained from 18 measurements. Following water refilling, four gas temperature drops of 3°C were observed and respective FRC recordings were subsequently excluded. Realistic breathing patterns were applied with V_T_ between 30 and 877 mL, V_T_/FRC between 0.14 and 0.55, and RR between 10 and 35 min^−1^. The intra-test variability of FRCs generated by the lung models was low overall, CV mean (SD) was 0.07 (0.31)%, and lower in the large model compared to the small model: CV mean (SD) was 0.03 (0.15)% and 0.23 (0.64)%, p = 0.108, respectively. The potential parallax error of reading water levels using the tape measure was estimated as one mm corresponding to 18.7 mL and 4.8 mL in the large and small lung model, respectively. Relating these volumes to the nominal FRC, the relative mean (range) parallax error was 1.2 (0.4–3.7)% and 2.9 (1.2–4.8)% in the large lung and small model, respectively.

### *In vitro* performance of the nitrogen multiple-breath washout setup

**Figure 2:**
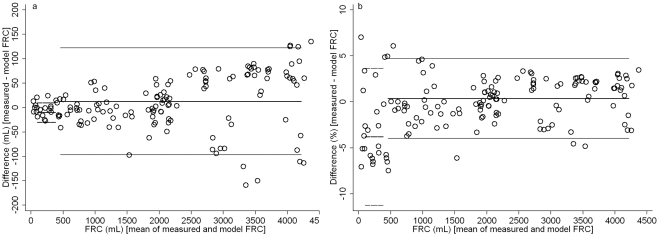
*In vitro* variability of functional residual capacity measurement. *Bland Altman* plot [16] of measured functional residual capacity (FRC) minus lung model FRC plotted vs. mean of measured and model FRC. Differences (open circles), and mean difference, and upper and lower limits of agreement (mean difference ±1.96 SD of differences) are given as dashed lines for the small lung model and as solid lines for the large lung model. Figure 2a gives absolute FRC differences (mL). For the small and large lung model, mean differences are −10.2 mL and 12.5 mL, upper limits of agreement are 9.9 mL and 121.9 mL, and lower limits of agreement are −30.3 mL and −96.8 mL, respectively. Figure 2b gives relative FRC differences (%). For the small and large lung model, mean differences are −3.8% and 0.4%, upper limits of agreement are 3.6% and 4.7%, and lower limits of agreements are −11.2% and −4.0%, respectively.

**Figure 3:**
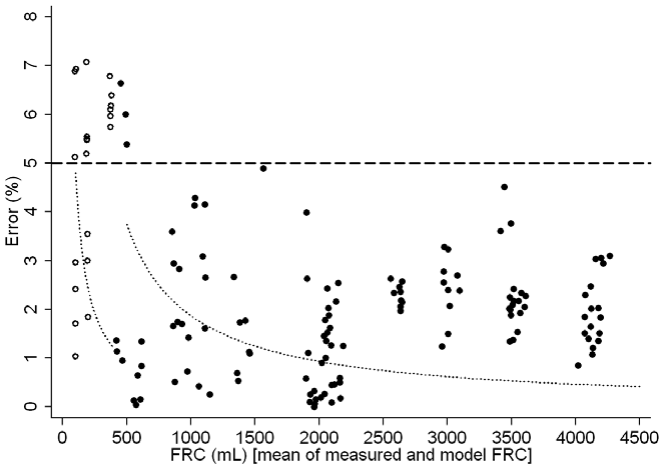
Association of measurement error with lung volume. Functional residual capacity (FRC) measurement error (%) is plotted against lung model FRC. The error data are displayed as open circles for the small model (100–400 mL FRCs) and closed circles for the large model (500–4200 mL FRCs). The dashed line gives the 5% limit of acceptable measurement error, the dotted lines reflect possible nominal volume bias due to reading parallax error (one mm) in the small and large lung model.

The O_2_, CO_2_, and N_2_ signals were linear over the full range. R^2^ was >0.99 for all signals (Table 2). The N_2_MBW setup accurately measured FRC with low intra-test variability (Figure 2, Table 3). Of 146 FRC measurements one measurement was excluded due to incomplete N_2_ washout. In 48 triplicate FRC measurements, mean (SD) of CV was 1.4 (1.7)%. Absolute mean (SD) difference between measured and model FRC was 9.2 (52.3) mL, relative difference was −0.2 (2.9)%. Of 145 FRCs measured between 100 mL and 4200 mL, 130 (90%) FRC measurements were within the 5% of model FRCs (Figures 2, 3). Mean (SD) error was 2.3 (1.7)% in all measurements. In the large lung model (500 to 4174 mL FRCs), 121/124 (98%) of FRCs were within the 5% error limit, error range was 0.0–6.5%. In the small lung model (100 to 400 mL FRCs), 9 (43%) out of 21 FRCs were within the 5% error limit, error range was 1.0–7.1%.

**Table 2:** Linearity of sensors.

	Offset		Gain		
	Mean	95% CI	Mean	95% CI	R^2^
**Oxygen** (%)	−0.47	−0.79 to −0.15	1.01	1.00 to 1.01	>0.99
**Carbon dioxide** (%)	−0.05	−0.12 to 0.02	1.08	1.06 to 1.10	>0.99
**Nitrogen** (%)	0.09	−0.17 to 0.35	0.99	0.98 to 1.00	>0.99

Oxygen and carbon dioxide and the derived nitrogen signals from the nitrogen multiple-breath washout setup were compared to mass spectrometer signals (AMIS 2000). Linear regression derived intercept (model offset), model slope (model gain) with respective 95% confidence intervals (CI), and R-squared (R^2^) are given.

**Table 3:** Nitrogen multiple-breath washout outcomes and variability.

*In vitro* nitrogen multiple-breath washout outcomes					
	Mean (SD)	CV (%)	Mean diff. (95% CI)^§^	CR	CR (%)
FRC^*^ (mL)	207.5 (107.0)	2.7 (2.0)	−10.2 (−5.5; −14.9)	20.1 mL	9.7%
**FRC^†^** (mL)	2360.0 (1186.1)	1.0 (1.4)	12.5 (2.6; 22.5)	109.3 mL	4.6%

*In vivo* nitrogen multiple-breath washout outcomes					
**FRC^‡^** (mL)	3249.6 (728.3)	4.5 (3.2)	−15.4 (−169.5; 138.7)	808.7 mL	24.9%
**LCI**	7.19 (0.53)	3.2 (1.7)	−0.13 (−0.26; 0.00)	0.68	9.5%
**S_acin_**	0.063 (0.029)	n.a.	0.001 (−0.005; 0.007)	0.031	49.2%
**S_cond_**	0.019 (0.005)	n.a.	0.000 (−0.002; 0.001)	0.008	41.4%

Triplicate nitrogen multiple-breath washout (N_2_MBW) tests applied to measure functional residual capacity (FRC) *in vitro* in the small^*^ (n = 7) and the large^†^ lung model (n = 41), and in 30 healthy adults^‡^. LCI = lung clearance index. S_acin_ = phase III slope index of *acinar* ventilation inhomogeneity. S_cond_ = phase III slope index of conductive ventilation inhomogeneity. CV = intra-test coefficient of variation (SD/mean*100). Mean difference^§^ (95% confidence interval) from paired *t*-tests comparing measured and actual model FRC [*in vitro*] or N_2_MBW outcomes between two test occasions [*in vivo*]. CR = coefficient of repeatability (1.96*SD of differences between measured and actual model FRC or differences of N_2_MBW outcomes between tests). CR is given in respective units or as percentage of the mean. n.a. = not applicable.

Errors in FRC measurements in lung models containing either ambient or CO_2_ enriched air were similar. Comparing the mean (SD) error using ambient air (n = 102) *vs.* CO_2_ enriched air (n = 43), the mean (SD) error was 2.2 (1.7)% *vs.* 2.6 (1.8)%, p = 0.185. Errors in FRCs measurements recorded on day one (n = 72) *vs.* day two (n = 73) differed, mean (SD) error 1.6 (1.0)% *vs.* 2.1 (1.4)%, p = 0.024. There was a positive association between error and RR (R^2^ = 0.24, p<0.001), and negative associations with V_T_ (R^2^ = 0.16, p<0.001), mean expiratory flow (R^2^ = 0.15, p<0.001), measurement duration (R^2^ = 0.12, p<0.001), and FRC (R^2^ = 0.09, p<0.001). Accounting for RR in a multi-variable regression model, the association of error with mean expiratory flow remained significant (p = 0.036) but not with V_T_, measurement duration, or FRC.

### *In vivo* performance of the nitrogen multiple-breath washout setup

Thirty out of 32 healthy adults (15 females) successfully performed N_2_MBW on two test occasions with a mean (SD) of 12.2 (5.1) days in between. Mean (range) age was 49.3 (21–69) years. Two males did not achieve leak-free N_2_MBW. All subjects had normal spirometry indices. Mean (SD) z-scores [17] of forced expiratory volume in one second (FEV_1_), FEV_1_/forced vital capacity, and forced expiratory flow between 25–75% of expired volume (FEF_25–75_) were 0.75 (0.86), 0.24 (0.81), and 0.32 (0.82), respectively.

**Figure 4:**
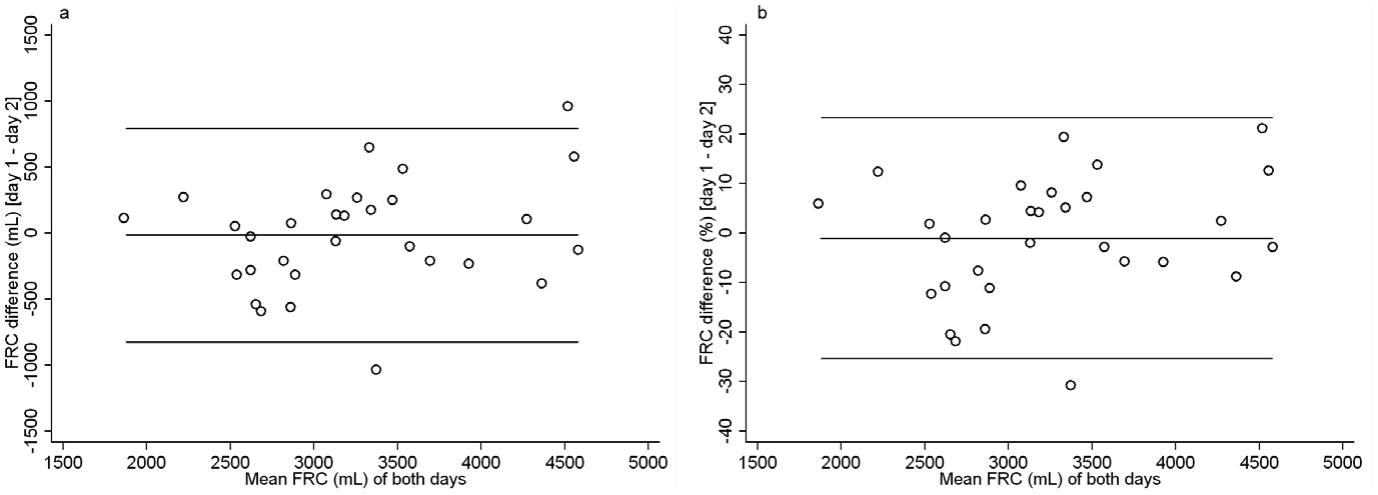
*In vivo* variability of functional residual capacity. *Bland Altman* plot [16] of functional residual capacity (FRC) measured on two study days within three weeks in 30 healthy adults. Absolute FRC differences (Figure 4a), relative FRC differences (Figure 4b), mean difference and upper and lower limits of agreement (lines) are plotted against mean FRC of both tests. Mean difference is −15.4 mL (−1.1%), upper and lower limits of agreement are 793.3 mL (23.2%) and −824.1 mL (−25.4%).

**Figure 5:**
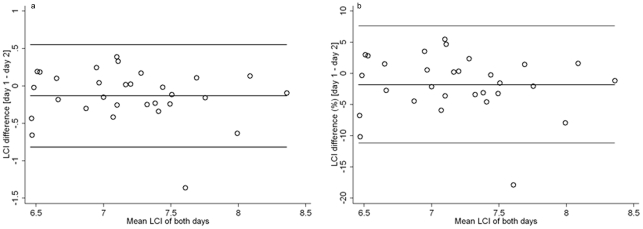
*In vivo* variability of lung clearance index. *Bland Altman* plot [16] of lung clearance index (LCI) measured on two study days within three weeks in 30 healthy adults. Absolute LCI differences (Figure 5a), relative LCI differences (Figure 5b), mean difference and upper and lower limits of agreement (lines) are plotted against mean LCI of both study days. Mean difference is −0.13 (−1.8%), upper and lower limits of agreement are 0.55 (7.6%) and −0.81 (−11.2%).

*In vivo* FRC intra-test variability was greater than measured in model FRC: CV mean (SD) was 4.5 (3.2)% *vs.* 1.4 (1.7)%, p<0.001. Likewise the *in vivo* FRC error between tests was greater compared to the *in vitro* FRC error measured in the models: Error mean (SD) was 10.1 (8.2)% *vs.* 2.3 (1.7)%, p<0.001. Reproducibility of ventilation inhomogeneity indices differed significantly with the LCI representing the most reproducible index (Table 3). *In vivo* reproducibility for LCI was better than for FRC, and markedly better than for S_acin_ and S_cond_ (Figures 4, 5). LCI was correlated with S_acin_ (*Pearson* r = 0.61, p<0.001) but not with S_cond_ (r = 0.17, p = 0.377). S_acin_ was correlated with S_cond_ (r = 0.44, p = 0.016). Estimates of repeatability (CV) and reproducibility (CR) of FRC and ventilation inhomogeneity indices were not associated with number of days between tests or the subject's age and gender (data not shown).

## Discussion

### Summary

This is the first study demonstrating that an available N_2_MBW system accurately and reliably measures FRC over the wide range of lung volumes and different breathing patterns encountered from early childhood to adulthood. This study also shows that BTPS conditions can be implemented into lung model studies to bench-test MBW equipment under realistic conditions. *In vitro* FRC can be measured with only 5% error over FRC volumes between 600 and 4200 mL with the N_2_MBW system. In FRCs below 600 mL, the error is better than 7%. N_2_MBW measurements in healthy adults show that LCI is more reproducible than FRC, S_acin_, and S_cond_. Variability of FRC measured *in vivo* is higher than *in vitro* suggesting substantial physiological fluctuation of FRC in humans [18].

### Comparison to previous lung model studies

Data on quality of signals and their integration under realistic conditions are essential for appropriate evaluation of MBW setups [19]. As an initial step, linearity of O_2_, CO_2_, and N_2_ signals was confirmed over the full measurement range in heated humid conditions encountered during clinical N_2_MBW testing. We improved a lung model described by *Brunner et al.* [20], which was originally ventilated manually and the water contained within the model was not heated. In addition to the incorporation of BTPS conditions, physiological breathing patterns were applied. These conditions recreate the stress the system is exposed to during clinical testing across wide age ranges. Previous *in vitro* lung models lacked one or more important physiological aspects [21]–[25].

The current N_2_MBW system precisely measures lung volumes between 600 and 4200 mL. In this volume range approximating late preschool age to large sized adults, the *in vitro* measurement error was below 5% in all FRCs (Figure 3). Importantly, this N_2_MBW system is not susceptible to different F_CO2_. Indirect N_2_ measurement may be susceptible to drift in F_CO2_ as the current N_2_ signal or, *e.g.* molar mass, are sum signals of gas fractions including the F_CO2_ [26].

This study is able to disentangle technical from physiological aspects contributing to FRC variability. Comparing the *in vitro* CR of FRC (4.6%) with *in vivo* (24.9%), inherent physiological variability of FRC may explain 20.3% (or relatively 82%) of between-test variability in adults. Validation studies exclusively performed *in vivo* do not allow differentiation between technical and physiological variability [24], [27]. In infants, the FRC error between tests may exceed 20% [28], [29].

### Ventilation inhomogeneity indices

The current N_2_MBW setup can be easily applied to measure ventilation inhomogeneity indices *in vivo*. These measures correlate as shown previously [15], [30]. LCI, in particular, had high repeatability and reproducibility in healthy adults. The specific indices of non-uniform gas mixing in the *conducting* and *acinar* airway zones (S_cond_ and S_acin_) were more variable. Between-test reproducibility of LCI (CR = 9.5%) was markedly higher than for S_acin_ (49.2%) and S_cond_ (41.4%). In a recent study [31], comparable between-test reproducibility of LCI, S_acin_, and S_cond_ was reported in healthy adults performing a sulfur hexafluoride MBW protocol with tidal breathing restricted to one liter V_T_. The small technical error of FRC observed *in vitro* strongly suggests higher physiological variability of these Sn_III_ indices compared to volume indices (FRC, LCI). Sn_III_ depends on breathing pattern [32], which was not restricted in this study. Interestingly, LCI is even more reproducible than FRC as also shown in healthy children [33]. This may reflect the fact that LCI is a robust volume ratio (CEV/FRC), cancelling the effect of physiological changes in lung volumes.

### Limitations

While the large lung model was suited for the MBW bench test, the small model may require adjustments. Automated ventilation and a laser sensor for determining nominal FRC could improve the infant lung model. Parallax error in the large model was small enough to avoid over- or underestimation of nominal FRCs (Figure 3).

Within the small technical measurement error of FRC, RR and tidal flow were correlated with error. Whether this association was causal leading to signal asynchrony or rather a surrogate for flow-dependent temperature and humidity fluctuations hampering adequate BTPS correction remains to be determined [10]. We hypothesize that the apparatus dead space volumes behave as systems taking up and delivering heat energy and moisture non-linearly over breath cycles. Reduced dead space and dynamic BTPS and synchronization algorithms may further decrease measurement error.

The current and previous models did not allow assessment of “tissue” N_2_ contribution to the lung inherent F_N2_. This contribution is difficult to estimate and account for, as available data are limited [34]. In adults with more advanced lung disease, duration of N_2_MBW and bias from tissue N_2_ may increase, and intervals between tests should be adapted accordingly [8].

### Implications

Availability of realistic validation protocols will aid the transition of MBW from promising research tool into routine clinical use. This is the first validation of an available N_2_MBW setup using physiological *in vitro* test conditions. Many other MBW setups remain to be validated and are either customized, expensive, or have been taken off the market [2], [3], [9], [35]. N_2_MBW is also advantageous to using foreign tracer gases because 100% O_2_ is economic, readily available in all hospitals, and non-polluting.

The between-test variability of ventilation inhomogeneity indices reported here sheds further light on their natural variability over time. The CR aids estimating clinically relevant change in lung function outcomes for future intervention studies. Given a hypothetical intervention in the current study, a decrease of LCI of 0.68 units (9.5%) would constitute a physiological change with 95% probability. Further longitudinal studies in disease groups, such as cystic fibrosis where variability is increased, are required [36]–[38]. In infants and preschool-aged children, safety of N_2_MBW and variability of N_2_MBW indices need to be determined.

### Conclusion

The performance of MBW setups can be realistically assessed using a new *in vitro* lung model producing BTPS conditions and representative breathing patterns. This lung model protocol is suitable for validation of other MBW systems. The new N_2_MBW system accurately and precisely measures lung volumes between 600 and 4200 mL. Future work may extend this into smaller lung volumes and younger age groups. The LCI represents the most reproducible N_2_MBW *in vivo* outcome as compared to FRC, S_cond_, and S_acin_. This study may aid the transition of this important type of lung function test from being a promising research tool to becoming a routine method in clinical practice.
